# Bacterial Genome Wide Association Studies (bGWAS) and Transcriptomics Identifies Cryptic Antimicrobial Resistance Mechanisms in *Acinetobacter baumannii*

**DOI:** 10.3389/fpubh.2020.00451

**Published:** 2020-09-02

**Authors:** Chandler Roe, Charles H. D. Williamson, Adam J. Vazquez, Kristen Kyger, Michael Valentine, Jolene R. Bowers, Paul D. Phillips, Veronica Harrison, Elizabeth Driebe, David M. Engelthaler, Jason W. Sahl

**Affiliations:** ^1^Northern Arizona University, Flagstaff, AZ, United States; ^2^Translational Genomics Research Institute, Flagstaff, AZ, United States

**Keywords:** genomics, transcriptomics, bioinformatics, acinetobacter, AMR

## Abstract

Antimicrobial resistance (AMR) in the nosocomial pathogen, *Acinetobacter baumannii*, is becoming a serious public health threat. While some mechanisms of AMR have been reported, understanding novel mechanisms of resistance is critical for identifying emerging resistance. One of the first steps in identifying novel AMR mechanisms is performing genotype/phenotype association studies; however, performing these studies is complicated by the plastic nature of the *A. baumannii* pan-genome. In this study, we compared the antibiograms of 12 antimicrobials associated with multiple drug families for 84 *A. baumannii* isolates, many isolated in Arizona, USA. *in silico* screening of these genomes for known AMR mechanisms failed to identify clear correlations for most drugs. We then performed a bacterial genome wide association study (bGWAS) looking for associations between all possible 21-mers; this approach generally failed to identify mechanisms that explained the resistance phenotype. In order to decrease the genomic noise associated with population stratification, we compared four phylogenetically-related pairs of isolates with differing susceptibility profiles. RNA-Sequencing (RNA-Seq) was performed on paired isolates and differentially-expressed genes were identified. In these isolate pairs, five different potential mechanisms were identified, highlighting the difficulty of broad AMR surveillance in this species. To verify and validate differential expression, amplicon sequencing was performed. These results suggest that a diagnostic platform based on gene expression rather than genomics alone may be beneficial in certain surveillance efforts. The implementation of such advanced diagnostics coupled with increased AMR surveillance will potentially improve *A. baumannii* infection treatment and patient outcomes.

## Introduction

Antimicrobial resistance (AMR) has the potential to become a global health emergency and is expected to kill more people than cancer by the year 2050 (1). Multidrug resistance in *Acinetobacter baumannii* is now recognized as a major public health concern, resulting in the World Health Organization (WHO) declaring *A. baumannii* a priority 1 pathogen (2). *A. baumannii* is primarily a nosocomial pathogen (3) that affects immunocompromised patients, causing a variety of afflictions including pneumonia, septicemia, meningitis, and death (4, 5). Treatment of *A. baumannii* infections has become increasingly difficult due to the emergence of multidrug resistance; pan-resistant *A. baumannii* strains (6–8), including strains resistant to last-resort drugs such as colistin (9), have been identified in Asia and Europe.

Known mechanisms that confer AMR in *A. baumannii* include penicillin binding proteins (10), enzymes (11), porin defects (12), 16S rRNA methylation (13), and efflux (14). Efflux pumps that confer resistance in *Acinetobacter* are classified into four families: multidrug and toxic compound extrusion (MATE), resistance–nodulation–division (RND) family, major facilitator superfamily (MFS), and small multidrug resistance (SMR) (15). Additionally, mutations in promoter regions can lead to overexpression of some efflux systems, including AdeFGH (14), which has been shown to lead to resistance to multiple antimicrobial families.

Resistance mechanisms have also been reported for specific drug families used to treat *A. baumannii* infections. Perhaps the most studied family is beta-lactams, including carbapenems (e.g., meropenem and imipenem), which are used to treat recalcitrant nosocomial infections (16, 17). Carbapenem resistance has been associated with the action of carbapenem-hydrolyzing class D beta-lactamases (CHDLs), including *bla*_OXA−23_, *bla*_OXA−24_, and *bla*_OXA−58_ (18). The *ampC* cephalosporinase is a class C beta-lactamase that is broadly conserved across *A. baumannii* (19) and has been associated with resistance to narrow spectrum cephalosporins (20). Additionally, *bla*_OXA−51−like_ genes are highly conserved across the *A. baumannii* species, as well as other *Acinetobacter* spp. (21); these genes confer resistance to carbapenems when in close proximity to the insertion element, IS*Aba1* (22).

Aminoglycoside resistance in *A. baumannii* has been associated with the actions of aminoglycoside modifying enzymes (AMEs) including *aacC1, aphA6, aadA1*, and *aadB* (23), the 16S rRNA methyltransferase *armA* (24), as well as through efflux action of AdeABC and AbeM, although the efflux effect is limited (23). Resistance to macrolides in *A. baumannii* has primarily been associated with target site alteration in Dfr (25) encoded by *folA*, the presence and activity of the *tetM* gene (26), and through the action of efflux pumps (27). Finally, quinolone resistance in *A. baumannii* has been linked to quinolone resistance-determining regions (QRDRs) (28), including mutations in *parC, gyrA*, and *gyrB*. Specifically, the *gyrA* S82L mutation has previously been shown to confer resistance to quinolones (29); two separate mutations, S83L and G80V, in *gyrA* have also been demonstrated to confer quinolone resistance in *A. baumannii* (30).

In recent years, multiple databases have been developed and maintained that include genomic regions associated with antimicrobial resistance. These databases include CARD (31), ResFinder (32), ARG-ANNOT (33), ARDB (34), and MEGARes (35). To identify potential resistance mechanisms, genomes are screened against these databases and if genes associated with resistance are identified, then resistance patterns are inferred (36). However, genomics doesn't capture expression profiles, including gene induction, which prevents accurate genotype to phenotype associations in some organisms (37).

Current treatment regimens for *A. baumannii* infections start with broad spectrum cephalosporins such as ceftazidime or cefepime, or a carbapenem (e.g., imipenem) (38). For drug resistant pathogens, polymyxins such as colistin are used, although emerging resistance has been reported (39) and the treatment can be toxic (40). Other drugs, including tigecycline (41) and minocycline (42) have been used to treat resistant strains, although resistance to these therapies has also been observed, prompting research into combination therapies (43) that overcome these limitations. However, pan-resistance in *A. baumannii* (44) has the potential to undermine all current treatment regimens and necessitates a better understanding of genotype/phenotype associations for improved surveillance efforts and targeted therapy.

Research into genotype/phenotype associations in *A. baumannii* is complicated by the highly plastic nature of the pan-genome (45). One example of this phenomenon is the biofilm associated protein (Bap) (46, 47), which, based on an *in silico* screen of more than 117 genomes annotated as *A. baumannii*, is found only in 2 genomes (unpublished). This demonstrates that mechanisms associated with a phenotype may not be broadly distributed across diverse isolates of this species. While true for virulence, similar patterns exist for AMR genes that are variably conserved within a highly plastic species (21).

In this study, we analyzed over 100 *Acinetobacter* isolates, largely isolated in Arizona, USA, in an effort to identify common as well as cryptic mechanisms of AMR. Implementing an iterative approach, we searched common AMR gene databases for known mechanisms, performed a bacterial genome wide association study (bGWAS) to identify potentially new mechanisms, and performed RNA-Sequencing (RNA-Seq) to compare gene expression profiles between isolates with variable AMR phenotypes. The results provide additional detail to understand AMR mechanisms in *A. baumannii* and identify targets for advanced diagnostics that will guide appropriate therapies for more effective patient treatment.

## Methods

### Isolate Description and Growth

A total of 107, largely geographically confined isolates, were identified for sequencing based on collection from different body sites and clinical matrices. All isolates were classified as *A. baumannii* based on orthogonal, clinical laboratory techniques. A description of all sequenced isolates is shown in Table S1. Samples were streaked from glycerol stocks onto Mueller Hinton (MH) (Hardy Diagnostics, Santa Maria, CA) agar plates and incubated at 37°C for 24 h. Inoculated plates were checked for appropriate colony growth and morphology the following day prior to DNA extraction.

### Genomic DNA Extraction and Sequencing

Genomic DNA was extracted from a single isolated colony for each sample using the DNeasy Blood and Tissue Kit (Qiagen, Valencia, CA, USA) following the recommended protocol for Gram-negative bacteria. Sample DNA was fragmented using the QSonica q800 ultrasonic liquid processor (QSonica, Newtown, CT, USA). Sonication parameters were optimized to produce fragment sizes of 600–700 base pairs (time: 3 min, pulse: 15 s (Pulse On), 15 s (Pulse Off), amplitude: 20%). Libraries were size selected using Agencourt AMPure XP beads (Beckman Coulter, Brea, CA) in order to remove small and large fragments outside of the required size range. Genome libraries were prepared using the KAPA Hyper Library Preparation Kit with Standard PCR Library Amplification (Kapa Biosystems, Wilmington, MA) and sequenced on an Illumina MiSeq using V3 sequencing chemistry (Illumina Inc., San Diego, CA).

For MinION sequencing, DNA was extracted with the GenElute Bacterial Genomic DNA kit (Sigma-Aldrich Inc., St. Louis, MO), taking care to limit DNA shearing. Long read sequencing was performed using Oxford Nanopore technologies on a MK1B MinION device using a R9.4 flow cell. The DNA library was prepared using the SQK-LSK109 Ligation Sequencing kit in conjunction with the PCR-Free Native Barcode Expansion kit following manufacturer's protocol (downloaded from https://nanoporetech.com/resource-centre/protocols/ on March 20, 2019) without the optional shearing steps to select for long reads.

### Sequence Assembly and MLST Typing

Illumina-derived whole genome sequence data was assembled with SPAdes v3.10 (48). Contigs that aligned against known contaminants or contained an anomalously low depth of coverage compared to the average depth of coverage on a per genome basis were manually removed. The MLST profiles were extracted from whole genome sequence (WGS) assemblies using BLAST-based methods (49) using both the Oxford (50) and Pasteur systems (51); novel alleles and sequence types were submitted to both databases. Annotation on all genomes was performed with Prokka v1.13 (52). Hybrid assemblies were generated with combined Illumina and MinION data with Unicycler v0.4.8-beta (53), which includes a polishing strep with Pilon v1.22 (54).

### Global Phylogenetics of *Acinetobacter* spp.

*Acinetobacter* genome assemblies were downloaded from GenBank on March 13th, 2018. All genome assemblies were aligned against the *A. baumannii* genome AB307-2094 (CP001172.1) with NUCmer v3.1 (55) in conjunction with NASP v1.1.2 (56). SNPs that fell within duplicated regions, based on a reference self-alignment with NUCmer, were filtered from downstream analyses. For rapid evaluation, an approximate maximum likelihood phylogeny was inferred on a concatenation of 1, 523, 968 single nucleotide polymorphisms (SNPs) with FastTree v2.1.8 (57); SNPs were retained if they were conserved in >90% of all genomes.

### Antimicrobial Resistance Profiling

Antimicrobial resistance phenotypic profiles were identified for cefepime (FEP), cefuroxime (CXM), gentamicin (GEN), ceftazidime (CAZ), trimethoprim (TMP), azithromycin (AZM), ceftriaxone (CRO), aztreonam (ATM), erythromycin (ERY), piperacillin (PIP), levofloxacin (LVX), imipenem (IPM), and ciprofloxacin (CIP). A list of all drugs and resistance breakpoints used are shown in Table 1. Drugs were selected from published resistance patterns in the literature (58–60, 62–69). Samples were streaked from glycerol stocks onto Mueller Hinton (MH) (Hardy Diagnostics, Santa Maria, CA) agar plates and incubated at 37°C for 24 h. A single isolated colony was picked and inoculated into 10 mL of MH broth. Liquid cultures were incubated with shaking at 37°C overnight. The following morning, 100 μL of each overnight culture were transferred into 9.9 mL of fresh MH broth. Cultures were incubated with shaking at 37°C until optical density (OD_600_) measurements reached 0.5–0.8, indicating log phase growth. Fifty microliter of culture was inoculated onto new 15 × 150 mm MH agar plates and spread uniformly across the medium with a sterile cell spreader. Six different antimicrobial E-test strips (bioMérieux, France) were applied to the surface of the agar as directed by the manufacturer. Plates were incubated at 37°C for 16–18 h and minimum inhibitory concentrations (MIC) were determined by visual inspection following the recommended manufacturer guidelines. For paired isolates, MIC tests were performed on different days. If the MIC was above CLSI guidelines for resistance, the strain was given an “R” (resistant) for that drug. If the MIC was below guidelines it was given an “S” (susceptible); if the MIC was between thresholds, it was given an “I” (intermediate). If biological replicates differed by more than one MIC dilution, the test was repeated. If replicates differed by a single MIC dilution, the lower MIC value was reported.

**Table 1:** Breakpoints of antimicrobials screened in this study.

**Antimicrobial**	**Abbreviation**	**Family**	**Publication**	**Resistant breakpoint**	**Susceptible breakpoint**	**References**
Cefepime	FEP	B-lactam	Endimiani et al. (58)	≥32	≤8	CLSI 2020
Cefuroxime	CXM	B-lactam	Ahmed et al. (59)	≥128	≤32	N/A
Gentamicin	GEN	Aminoglycoside	Hamidian et al. (60)	≥16	≤4	CLSI 2020
Ceftazidime	CAZ	B-lactam	Lee et al. (61)	≥32	≤8	CLSI 2020
Trimethoprim	TMP	Pyrimidine inhibitor	McCracken et al. (62)	≥32	≤4	EUCAST 2018^a^
Azithromycin	AZM	Macrolide	Fernandez Cuenca et al. (63)	≥256	≤8	N/A
Ceftriaxone	CRO	B-lactam	Bush et al. (64)	≥64	≤8	CLSI 2020
Aztreonam	ATM	B-lactam	Xia et al. (65)	≥32	8	CLSI 2014
Erythromycin	ERY	Macrolide	Damier-Piolle et al. (66)	≥8	≤0.5	CLSI 2020^b^
Piperacillin	PIP	B-lactam	Shi et al. (67)	≥128	≤16	CLSI 2020
Levofloxacin	LVX	Fluroquinolone	Lee et al. (61)	>1	≤0.5	EUCAST 2020
Ciprofloxacin	CIP	Fluroquinolone	Chiu et al. (68)	≥4	≤1	CLSI 2020
Imipenem	IPM	B-lactam	Choi et al. (69)	≤2	≥8	CLSI 2020

a *Enterobacteriaceae*.

b *Enterococcus*.

### *A. baumannii* Phylogeny and Isolate Pairing

Once the confirmed set of *A. baumannii* were identified, a phylogeny was generated for 84 genomes. Raw WGS data were aligned against AB307-0294 with BWA-MEM v0.7.7 (70) and single nucleotide polymorphisms (SNPs) were identified with the UnifiedGenotyper method in GATK v3.3.1 (71, 72). SNPs that fell into duplicate regions of the reference, based on a NUCmer self-alignment, were removed from downstream analyses. All SNP calling methods were wrapped by the NASP pipeline. A maximum likelihood phylogeny was inferred on a concatenation of 182,766 SNPs with IQ-TREE v1.6.1, using the TVMe + ASC + R5 model. Paired genomes were identified by low phylogenetic distance and variable MIC profiles (Table S2).

### Global Phylogenetic Analysis *of A. baumannii*

All *A. baumannii* genomes (*n* = 3,218) were downloaded from the Assembly database in GenBank (73) on September 19th, 2018. Genomes were filtered if they: (1) contained >200 ambiguous nucleotides (*n* = 860); (2) contained >400 contigs (*n* = 189); (3) had a genome assembly size <3, 684, 234 or >4, 297, 137 (*n* = 51), or; (4) had an average MASH (74) distance >0.0252 (~97.5% average nucleotide identity) (*n* = 20). Genomes passing through all filters (*n* = 2183) were aligned against *A. baumannii* AB307-2094 (6) with NASP in conjunction with NUCmer. A maximum likelihood phylogeny was inferred on a concatenation of 101,608 SNPs with IQ-TREE v1.6.1, using the TVM+F+ASC+R10 model, and rooted with an *A. nosocomialis* genome sequenced in this study (TG22170; RFEG00000000); the smaller number of SNPs is due to the reduced size of the core genome due to the inclusion of fragmented genome assemblies in GenBank.

### Comparative Analysis of Paired Isolate Genomes

To identify coding region differences between paired isolates, the large-scale blast score ratio (LS-BSR) (75) tool was run on paired genomes in conjunction with BLAT (76). The order of genes between isolates was visualized with genoPlotR (77). The conservation of genes was compared between resistant and susceptible groups using a Mann-Whitney test implemented in Scipy v1.4.1 (78) and significance was applied at an alpha of 0.01.

### Bacterial Genome Wide Association Study (bGWAS)

To identify genotype/phenotype associations, regions identified by LS-BSR were compared between resistant/susceptible phenotypes from each drug. Regions were first identified that had a blast score ratio (BSR) value (79) of >0.8 in one phenotype and a BSR value of <0.4 in the other phenotype. The BSR is calculated by dividing the query Blast bit score by the self-Blast bit score; a BSR value of 0.8 is equivalent to 80% identity over 100% of the alignment length. In addition to bulk differences in coding region sequences (CDSs), differences between individual SNPs and indels were identified through the analysis of Kmers. In this approach, the reverse complement was taken for all genome assemblies so that both strands were included in the analysis. All 21-mers were then identified with Ray-surveyor (80) and placed into a presence absence matrix; the choice of 21-mers was to ensure a short enough length to hopefully identify single mutations. The frequency of Kmers in each phenotype was then calculated with a custom Python script (https://gist.github.com/jasonsahl/e9516b2d940ad2474ba6e97f5b856440) and significance was assessed with a Mann-Whitney test implemented in Scipy with a Bonferroni *p*-value correction. In addition to these analyses, SNPs associated with resistance phenotypes were identified with default parameters in treeWAS v1.0 (81).

From the raw sequence data, all possible 54-mers at a minimum frequency of 4 per genome were also processed with pyseer (82). A similarity matrix was generated with pyseer and included in the analysis to account for population structure. Pyseer was run using default settings, a similarity matrix, and a “maf” value of 0.40. Only Kmers with good chi-square values were included and were grouped by locus tag.

### Machine Learning

Three machine learning models were tested within the scikit-learn python module (83): gradient boosting classification (GBC), random forest (RF), and penalized logistic regression (LR). Parameters were tuned for each model over 1,000 50–50% train-test splits. For each antimicrobial studied, all three models were analyzed on the same 500 replicates of 75–25% train-test split cross validation. Each iteration began with a random seed to ensure different train-test splits.

### *In silico* Screen of Antimicrobial Resistance-Associated Genes

To identify the conservation of previously characterized antimicrobial resistance mechanisms, we screened all genomes against proteins from the Comprehensive Antimicrobial Resistance Database (CARD) (31) with LS-BSR in conjunction with Diamond (84).

### Antimicrobial Exposure and RNA Extractions

Samples identified as paired isolates based on phylogenetic relatedness and differing AMR susceptibility profiles were streaked for isolation from glycerol stocks onto MH agar plates and incubated overnight at 37°C. For each sample a single colony was picked and inoculated into 10 mL of MH broth and incubated with shaking at 37°C overnight. The following morning 100 μL of each culture was inoculated into 9.9 mL of fresh media and OD_600nm_ was monitored until cultures reached log phase growth OD_600nm_ of ~0.5–0.8. Five-hundred microliter of each sample, as well as the susceptible control strain, *Staphylococcus aureus subsp. aureus* (ATCC 29213), were aliquoted into 2 mL microcentrifuge tubes. Each sample was treated with sub-MIC concentrations of the designated antimicrobial, at one half of the previously recorded MIC value. Cultures were then incubated for 30 min with shaking at 37°C. Two volumes of RNAprotect Bacteria Reagent (Qiagen, Valencia, CA, USA) were added to all samples and incubated at room temperature for 5 min, followed by centrifugation for 10 min at room temperature, at a speed of 5,000 × g. The supernatant was decanted and the treated cell pellets were stored at −80°C. Total RNA was extracted using the RNeasy Mini Kit (Qiagen, Valencia, CA, USA) following recommended protocol #4 beginning at step 7 and continuing to protocol #7. A DNase I treatment was included for step 2 in protocol #7. Extracted RNA was immediately stored at −80°C. All mRNA extractions were performed in biological triplicate with cultures grown on separate days.

### mRNA Isolation

RNA quality and quantity were checked by Agilent 2100 Bioanalyzer with the RNA 6000 Nano Kit (Agilent Technologies, Santa Clara, CA, USA). mRNA was isolated from total RNA using the MICROBExpress kit (Thermo Fisher Scientific, Waltham, MA) following the manufacturer's protocol. Isolated mRNA was quantified and checked for rRNA depletion on the bioanalyzer with an additional RNA Nano chip prior to sequencing.

### RNA-Seq Preparation, Sequencing, and Assembly

Previously isolated mRNA was prepared for transcriptome sequencing using the TruSeq Stranded mRNA, HT kit (Illumina, San Diego, CA) following the High Sample (HS) protocol. Prepared samples were quantified and checked for quality, then pooled in equimolar concentrations. Library pools were loaded into an Illumina High Output NextSeq 2 × 150bp kit, according to manufacturer recommendations for sequencing on the Illumina NextSeq 550 platform. The transcriptomes were assembled with metaSPAdes (85) using default settings. For targeted amplicon studies, complementary DNA (cDNA) was generated with the SuperScript IV VILO RT-PCR Master Mix with ezDNase enzyme (Invitrogen, Carlsbad, California), following manufacturer's recommendations.

### Differential Expression (DE) Analysis

For each isolate pair, coding and intergenic regions identified with LS-BSR and prodigal were combined for complete genomes, then dereplicated with USEARCH v10 at an ID of 0.98. RNA-Seq reads were aligned against these regions with BWA-MEM and read counts were called on the resulting BAM file with Salmon v0.13.1 (86). Differential expression (DE) analysis was performed with DESeq2 (87). The *p*-values were corrected using the Benjamini-Hochberg (88) correction. For each pair, the resistant strain expression, including those samples grown in sub-MIC concentrations of antimicrobials, were individually compared to the expression of the susceptible strain.

### Amplicon Sequencing (AmpSeq)

Polymerase chain reaction (PCR) primers were designed for differentially expressed regions identified in the RNA-Seq analysis (Table S3); a constitutively expressed target (locus tag: IX87_18340), based on analysis of RNA-Seq data, was included for normalization. cDNA was amplified with the following protocol: 1X Promega PCR Master Mix (Promega, Fitchburg, WI), 2.5 μL cDNA template, and multiplexed primer concentrations listed in Table S3. Gene specific PCR parameters were as follows: initial denaturation at 95°C for 2 m, 30 cycles of denaturation at 95°C for 30 s, annealing at 55°C for 30 s, and extension at 72°C for 45 s, with a final extension at 72°C for 5 m. Included on each primer was a universal tail (89), which facilitated Illumina index ligation. Samples were indexed with the following final concentrations: 1X HiFi HotStart Readymix (Kapa Biosystems Inc., Wilmington, MA), 0.4 μM of each indexing primer, and 2 μL of gene specific PCR product. The indexing PCR parameters were as follows: initial denaturation at 98°C for 2 m, 6 cycles of denaturation at 98°C for 30 s, annealing at 60°C for 20 s, and extension at 72°C for 30 s, with a final extension at 72°C for 5 m. Following each PCR, a 1X Agencourt AMPure bead (Beckman Coulter, Brea, CA) clean-up was performed according to manufacturer's instructions. All amplicons were normalized with SequalPrep (Thermo Fisher Scientific, Applied Biosystems), pooled, and sequenced on the Illumina MiSeq platform (Illumina Inc., San Diego, CA).

### AmpSeq Analysis

Raw AmpSeq data were aligned against predicted amplicons with Kallisto v0.45.0 (90); predicted amplicons were identified by the *in silico* extraction of sequence between matching primer pairs. Counts were normalized based on the median read counts between all samples. The difference, or delta, between the raw read counts of the target and a constitutively expressed reference housekeeping gene was identified for each sample. The average delta was then identified for each set of resistant and intermediate genomes and the delta Ct was calculated. The average deltas were compared between resistant and intermediate samples and a *p*-value was calculated with a Mann-Whitney U test.

### Data Availability

All data were deposited to appropriate databases and linked under BioProject PRJNA497581. Accession information for specific samples is shown in Table S1.

## Results

### Identification of Isolates Analyzed in the Current Study

**Figure 1:**
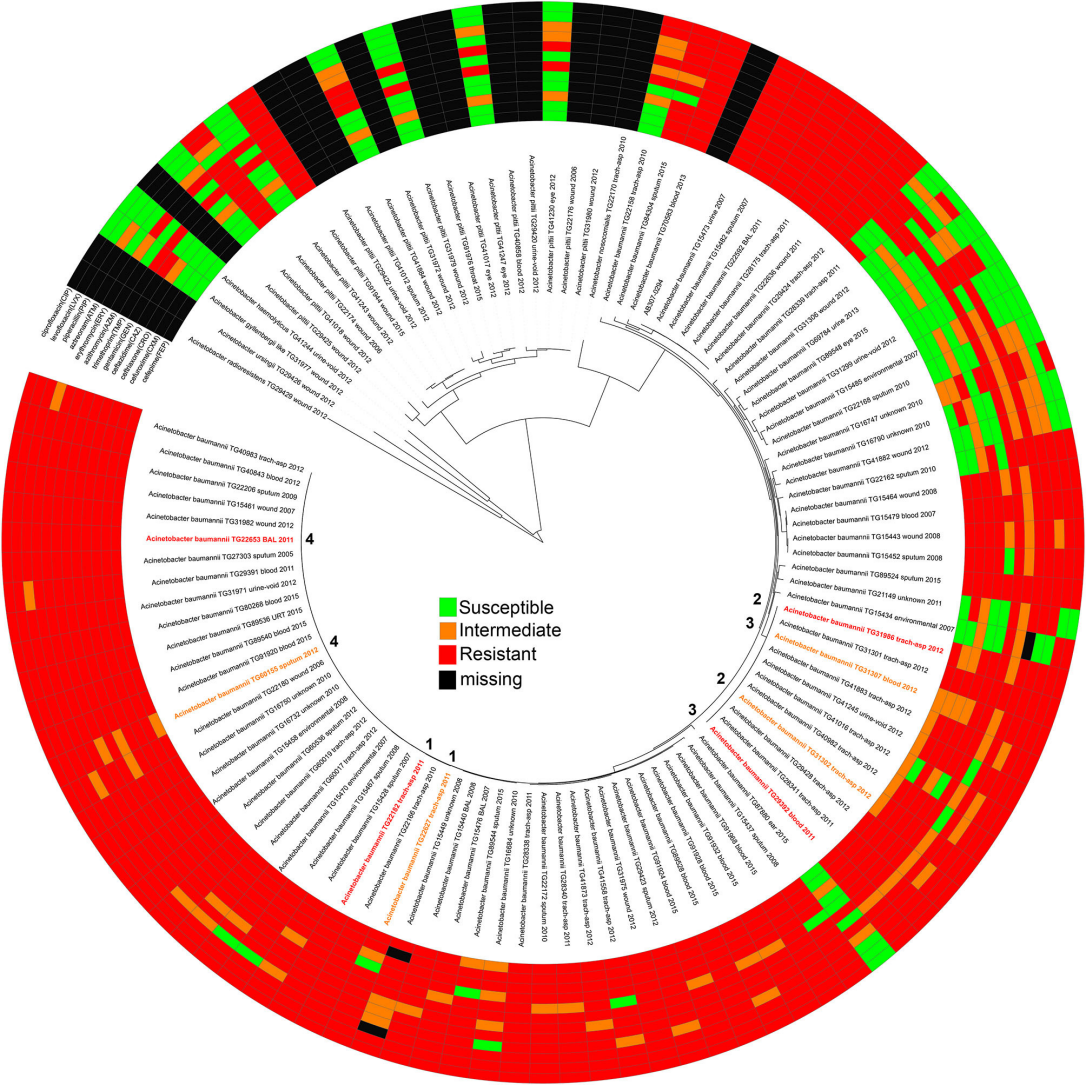
A maximum-likelihood phylogeny of *Acinetobacter* genomes sequenced in this study based on a concatenation of 50, 869 core genome SNPs. Each genome is annotated with its antimicrobial susceptibility profile across 12 drugs. The annotations were visualized with the Interactive tree of life (91). The pair information (1–4) is shown in the middle of the phylogeny.

In this study, we sequenced DNA from 107 isolates identified by laboratory methods to be *A. baumannii*. These isolates were retrospectively identified from our collection and sequenced to reflect a range of years and isolation sources (Table S1). Of the 107 genomes sequenced in this study, only 84 were confirmed *A. baumannii* (Table S1) isolates based on a global WGS phylogenetic analysis (Figure 1). In order to define and add context to the phylogenetic diversity of genomes sequenced in this study, more than 3,000 publicly available *A. baumannii* genomes were included in the analysis (Figure S1).

### Antimicrobial Resistance Profiles of Isolates Analyzed

AMR profiles were identified for 95 of the isolates across 12 drugs, including all *A. baumannii* (Figure 1, Table S2). Twelve isolates were excluded due to either difficult to interpret MIC results or inconsistent results across replicates. Some test strips were discontinued during the course of this experiment and were therefore marked as missing in the AMR profile. Although not analyzed further, the AMR profiles for non-*baumannii* genomes may be useful for researchers studying emerging resistance in other hospital-associated *Acinetobacter* species.

Antibiograms were obtained for the majority of isolates across tested drugs (Table S2) using E tests; selected drugs were chosen based on treatment suggestions in previous publications (58–68). The MIC values were mapped against a phylogeny of *A. baumannii* genomes (Figure 1) inferred from a concatenation of 182,916 SNPs. Resistant, susceptible, or intermediate calls were determined based on identified breakpoints (Table 1). Four of the drugs used in this study do not have an identified breakpoint for *Acinetobacter*. Breakpoints were applied for two of these drugs based on other organisms. For two additional drugs, breakpoints were applied that only includes the highest and lowest values of the E test range. This conservative approach is potentially useful for grouping isolates into categories to identify mechanisms associated with the largest differences in MIC values, but may not be clinically relevant.

From the *A. baumannii* phylogeny, isolates were identified that were closely related based on phylogenetic distance, but differed in their antibiograms. These isolate pairs (Figure 1, Table 2) were the subject of additional investigation in order to identify cryptic resistance mechanisms based on a common genomic background.

**Table 2:** Paired isolate antimicrobial susceptibility.

**Intermediate isolate**	**Resistant Isolate**	**Drug**	**Pair number**	**PubMLST/Pasteur**	**Resistant MIC**	**Other MIC**
TG22627	TG22182	CRO,CAZ,IPM	1	ST368/ST2	>256, >256, 16	48, 8, 4
TG31302	TG31986	FEP	2	ST1961/ST78	>256	12
TG31307	TG29392	CXM,CRO	3	ST1961/ST78	>256, >256	64, 32
TG60155	TG22653	FEP	4	ST208/ST2	>256	16

### *In silico* Screening of Paired Isolate Genomes

The 84 confirmed *A. baumannii* genomes were screened for the presence of AMR-associated genes from the CARD database with LS-BSR (Table S4). For 2 isolate pairs, no obvious differences were observed in resistance genes between variably resistant pairs (Figure S2, Table S5). For TG22182 (R) and TG22627 (I), one CARD gene was differentially present (aminoglycoside phosphotransferase (CAE51638)], although no differences were observed in resistance to the tested aminoglycoside, gentamicin; these genes potentially confer resistance to different aminoglycosides that were not screened in this study. Multiple differences were observed between the distribution of CARD genes between TG22653 (R) and TG60155 (I) (Figure S2), although the antibiograms only differed in the resistance to two antimicrobials and the genomes differed by only 27 core genome SNPs.

### *In silico* AMR Profiling of All Sequenced *Acinetobacter* Isolates

**Figure 2:**
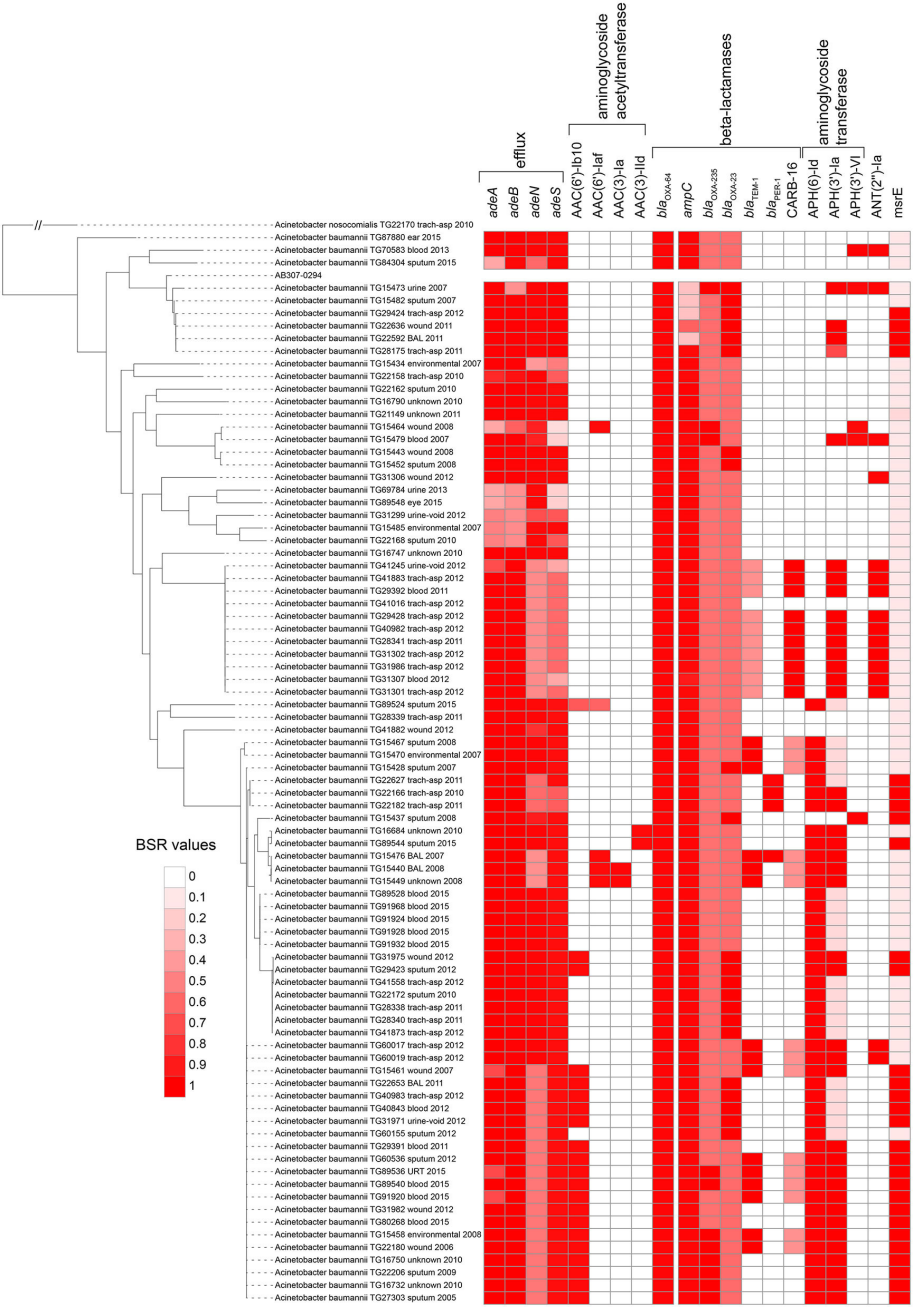
Screen of selected CARD proteins (31) across all *Acinetobacter baumannii* genomes sequenced in this study. The heatmap is associated with the blast score ratio (BSR) (79) values of each gene across each genome. The BSR values were visualized with the Interactive tree of life (91).

All proteins from the CARD database (*n* = 2,420) were aligned against 84 sequenced *A. baumannii* genomes. Proteins that were highly conserved in at least 2 genomes demonstrate variable conservation of AMR-associated proteins (Figure 2). Some proteins had a clear phylogenetic distribution, where they were either found across almost all *Acinetobacter* [e.g., *bla*_OXA−64_ (OXA-51 family)], conserved across phylogenetic groups in *A. baumannii* [e.g., aminoglycoside resistance genes (APH(6)-Id): AAC23556.1], or were variably present [e.g., aminoglycoside adenyltransferase (ANT(2”)-Ia): AAC64365.1], suggesting horizontal gene transfer.

### *In silico* Detection of Carbapenemase Genes

In addition to *bla*_OXA−64_, the beta-lactamase genes *bla*_OXA−23_ (92) and *bla*_OXA−235_ (93) were identified across a subset of sequenced *A. baumannii* genomes (Figure 2, Table S4). The extended spectrum beta lactamase *bla*_TEM−1_ (94) was present in 15 of 84 of sequenced genomes. Finally, 10 genomes contained a CARB-family (CARB-16), class A beta-lactamase (95). Imipenem was only screened across paired isolates in this study and 3 isolates were resistant based on established breakpoints (Table 1). Two of these resistant genomes (TG60155, TG22653) contained *bla*_OXA−23_, while 3 susceptible isolate genomes contained CARB-16. One resistant isolate genome (TG22627) was negative for all screened carbapenemase genes with the exception of *bla*_OXA−64_.

### Screen of Previously Described AMR Mechanisms

In addition to differentially-present CARD genes (Table S4), genomes were also screened for mechanisms associated with resistance to the following specific drugs:

#### Quinolones

The *gyrA* S82L mutation has previously been shown to confer resistance to quinolones in *Acinetobacter* (29). Of the resistant *A. baumannii* strains (*n* = 73), 72 (~99%) contained the leucine (L) residue at position 82; the one exception was TG22162, which had the serine (S) residue. All susceptible strains had the serine residue at this position, suggesting that this mutation is the primary mechanism conferring quinolone resistance in analyzed strains.

#### Trimethoprim

All tested *A. baumannii* isolates were resistant to trimethoprim. An *in silico* screen of *folA*, which has been associated with target site alteration and trimethoprim resistance (25), demonstrated that all genomes contained this gene, although there was some variation in the peptide identities (Table S4). As susceptible strains were not identified through screening, the genotype/phenotype relationship for this compound cannot be tested, although based on published results, *folA* appears to be the associated mechanism.

#### Beta-Lactams

The *ampC* gene in *A. baumannii* is a class C beta-lactamase (96). A screen of the *ampC* peptide sequence against *A. baumannii* isolates sequenced in this study indicates that almost all genomes have a highly conserved *ampC* gene (EA665_005650) (Figure 2, Table S4), but have widely different antibiograms (Table S2). A comparison of the *ampC* gene identified that a significant genotype/phenotype relationship was only observed for cefepime (FEP) (*p* = 0.006). This demonstrates that the presence/absence of this gene alone is not broadly associated with resistance to beta-lactams screened in this study.

The insertion element IS*Aba1*, in conjunction with *bla*_OXA−51−like_ genes, has been shown to confer resistance to beta-lactams (22). There was a significant difference in the conservation of IS*Aba*1, based on a Mann-WhitneyU test of BSR values for resistant and susceptible genomes, for 5 beta-lactams (cefepime, cefuroxime, ceftazidime, piperacillin, ceftriaxone) (*p* < 0.01). Many of the IS*Aba1* transposases were split across multiple contigs in Illumina assemblies, likely due to a repeat region that could not be resolved during assembly; this limited our ability to determine the proximity of IS*Aba*1 to the *bla*_OXA−51−like_ genes. Furthermore, copies of IS*Aba*1 were likely collapsed during the short read assembly. For example, for the 8 isolates for which draft genomes and complete genomes were generated in this study, only a single copy of IS*Aba1* was observed in each draft genome, while 9–26 copies were observed in complete genomes. Additionally, all of the paired isolates in this study contained IS*Aba1* and a *bla*_OXA−51−like_ gene but showed variable antibiograms to at least one beta-lactam, suggesting that the conservation of this region alone did not explain the resistance phenotype in the absence of proximity information.

Genes associated with efflux (*adeA, adeB*) were missing from six genomes (Figure 2, Table S4) that showed susceptibility to a number of antimicrobials including cefepime, cefuroxime, ceftazidime, and gentamicin (Table S2). Other genomes contained these genes but were also susceptible to beta-lactams, suggesting multiple genotypes result in the same resistance or susceptibility phenotype.

#### Macrolides

Although *ermB* has been associated with macrolide resistance in *A. baumannii*, the gene was not detected in any genome sequenced in this study, based on a LS-BSR analysis (Table S4). The *mefA* gene was also screened, as it has been demonstrated to provide macrolide resistance, but the gene (Table S4) was present in all 8 azithromycin susceptible strains (Table S2). Resistance to macrolides has also been associated with efflux, although differences in efflux cannot be investigated with genomics alone.

#### Aminoglycosides

CARD genes associated with aminoglycoside resistance were screened against genomes with LS-BSR. Of the 4 regions [AAC(3)-Ia, APH(3′)-VIa, *aadA1, aadB*] previously associated with aminoglycoside resistance, AAC(3)-Ia and *aadA1* both showed a significant association (*p* < 0.01) with gentamicin resistance in genomes screened in this study.

### Bacterial Genome Wide Association Study (bGWAS)

To identify genotype/phenotype associations, a bGWAS analysis was performed by splitting up isolates into resistant/susceptible groups for each drug based on defined thresholds (Table 1); for this analysis, isolates with an intermediate phenotype were ignored in order to isolate the mechanism. Using gene conservation, we failed to identify a clear genotype/phenotype relationship across all *Acinetobacter* across all drugs. This suggests that genomic analyses alone can fail to comprehensively identify AMR mechanisms in *Acinetobacter*. When the analysis was repeated for only *A. baumannii* genomes using genes, SNPs, and Kmers, significant associations were identified (Table 3, Table S6). The method testing genes and Kmers did not consider population structure, suggesting that the significance of these hits cannot be separated from population differences. TreeWas does incorporate population structure data and identified four SNPs associated with two different antimicrobials (gentamicin, levofloxacin). There was only one SNP that was associated with a non-synonymous mutation in a gene annotated as a rRNA methyltransferase. Ribosomal methylation has been demonstrated to confer resistance to aminoglycosides in multiple bacterial genera, including *Acinetobacter* (13).

**Table 3:** Differences in Kmers, genes, and SNPs between resistant (R) and susceptible (S) isolates.

**Drug**	**#Resistant isolates (R)**	**#Susceptible isolates (S)**	**#Associated Kmers (R)**	**#Associated Kmers (S)**	**#Associated genes (R)**	**#Associated genes (S)**	**#Associated SNPs**
ATM	57	7	0	0	0	0	0
AZM	50	6	0	0	0	0	0
CIP	73	11	0	0	0	0	0
ERY	81	0	N/A	N/A	N/A	N/A	N/A
GEN	57	16	0	0	0	0	10
LVX	71	12	0	0	0	0	2
FEP	61	13	0	0	0	0	0
PIP	70	5	0	3	0	0	0
CRO	67	3	3^*^	47	1^*^	0	0
CAZ	68	9	0	0	0	0	0
CXM	67	11	0	0	0	0	0

* *Present in n-1 genomes*.

For ceftriaxone (CRO), all susceptible strains (*n* = 3) were missing IS*Aba1* (ABLAC_32600), while 66 of 67 resistant strains contained this region; the small number of genomes analyzed limits the power of this analysis. In spite of this correlation, the lack of broadly conserved genomic regions associated with resistance directed a paired genome analysis into the identification of novel or cryptic genotype/phenotype associations.

An analysis with pyseer was also performed and found associated locus tags for 6 antimicrobials (Table S6). The locus ABBFA_01412, coding for a MFS transporter, was identified as significant by both TreeWas and pyseer. Pyseer also found a significant association of *parC* (ABBFA_03297) with resistance to levofloxacin and gentamicin; the association of levofloxacin resistance with mutations in *parC* have been observed previously in *Acinetobacter* (97) and may synergize with observed mutations in *gyrA* that were not identified by pyseer. For cefuroxime resistance, pyseer identified 3 Kmers that were significantly associated with *ampC* (ABBFA_001076). Although pyseer failed to identify a significant association with ISAba1, the *ampC* results suggests that the co-location of these regions is likely associated with the resistance phenotype.

### Machine Learning bGWAS Approach

Machine learning approaches were applied to a gene presence/absence matrix generated with LS-BSR. The results demonstrate that associations were identified for 7 of the tested antimicrobials (Table 4); the different machine learning algorithms found different regions associated with the resistance phenotype, designated by “N/A” in terms of rank of importance. While none of these genes can uniformly discriminate between resistant and susceptible genotypes, they may add to the resistance phenotype and could be the subject of additional investigations.

**Table 4:** Machine learning results.

**Drug**	**Locus**	**Protein product**	**Variable strength**	**Average BSR (Resistant)**	**Average BSR (susceptible)**
ATM	EA714_RS07565	hypothetical protein	0.91	0.75	0.41
CIP	EA712_RS18090	Outer membrane protein	0.68	0.94	0.26
GEN	EA737_RS20310	APH(3′)-Ia	0.58	0.66	0
LVX	EA737_RS18895	IS26 transposase	0.12	0.61	0.02
FEP	EA737_RS18895	IS26 transposase	0.29	0.64	0.02
PIP	EA728_RS02360	glycosyltransferase	0.11	0.96	0
CXM	EA717_RS18695	phage protein	0.13	0.88	0.3

### RNA-Seq and Differential Expression (DE) Analysis

For isolate pairs where a clear genotype/phenotype relationship was not identified, RNA was extracted and cDNA was sequenced. Despite implementing methods to enrich mRNA, ~20% rRNA + tRNA presence was observed in all samples. For each pair, all coding and intergenic sequences were combined into a single file and de-replicated. Reads were mapped against these regions, normalized, and differential expression was identified using DESeq2. Results were then identified for the following isolate pairs:

#### Pair 1

**Figure 3:**
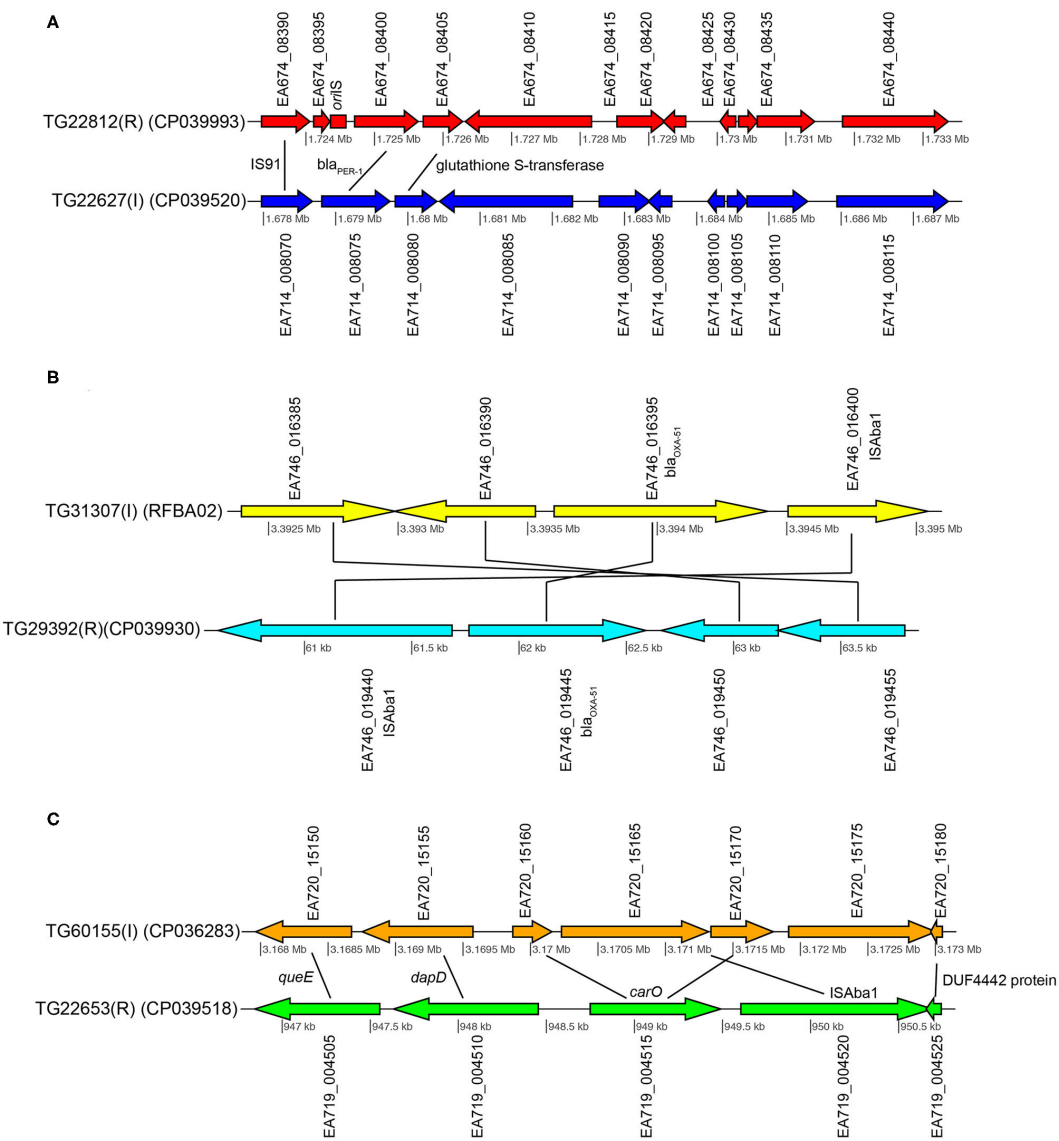
Gene content comparisons between paired isolates in pair 1 **(A)**, pair 3 **(B)**, and pair 4 **(C)**. All figure panels were generated with genoPlotR (77).

TG22627 (I) and TG22182 (R) showed variable susceptibility to ceftriaxone (CRO), imipenem (IPM), and ceftazidime (CAZ). Sixty-eight differentially expressed genes were identified that were upregulated in TG22182 (Table S7) using a Wald stat threshold of 10; this threshold was used to prioritize targets for further investigation, but may not capture all biologically-relevant differential expression. One of these regions (Table 5) was a PER-1 beta-lactamase gene (*bla*_PER−1_) (EA714_008075) that is not broadly conserved across sequenced genomes (Table S4) and appears to be present on a transposon. A screen of this gene against ST368 genomes isolated from diverse geographic locations suggested that there was an acquisition of this region in a single sequence type and a clear phylogenetic effect (Figure S3). Indeed, a genomic island was identified in both the resistant and intermediate genomes between two transposases (Figure 3A) that includes a Glutathione S-transferase gene (EA674_08405) that was also upregulated in the resistant strain (11.2x up-regulation); this region has previously been associated with beta-lactam resistance (98). The operon structure was similar between resistant and intermediate strains, with the exception of an IS91 transposon situated between an IS26 transposon and *bla*_PER−1_. The operon structure for the resistant strain that contained an IS91 transposon was determined to be highly similar with an ISCR1 (Insertion sequence Common Region) element. Within the ISCR1 element is an ori*IS* (origin of replication) region that allows for rolling-circle replication and transposition of the ISCR1 element. Within the ori*IS* are *two* outward-oriented promoters (P_OUT_) that have been shown to affect downstream gene expression (99). The resistant strain, TG22182, has both P_OUT_ promoters associated with increased gene expression directly upstream of the *bla*_PER−1_ gene (Figure 3A). The more susceptible strain, TG22627, has neither of the P_OUT_ promoters upstream of its *bla*_PER−1_ gene. Additionally, the composition of the *bla*_PER−1_ gene between isolates was different (Table 5), with a different coding length as well as composition in the first 12 amino acids of the peptide.

**Table 5:** Conservation and expression of differentially-expressed loci between resistant (R) and intermediate (I) strains.

**Pair**	**Locus**	**Product**	**Genome BSR (R)**	**Genome BSR (I)**	**Average Counts (R)**	**Average Counts (I)**	**Wald stat**
1	EA674_08405	Glutathione S-transferase	1.00	1.00	3, 331	349	42.62
1	EA674_03600	Multidrug efflux permease AdeJ	1.00	1.00	1, 800	17, 112	41.74
1	EA674_03605	Multidrug efflux transporterAdeI	1.00	1.00	651	7, 275	39.04
1	EA674_08405	Glutathione S-transferase	1.00	1.00	3, 834	349	42.62
1	EA714_008075	PER family beta-lactamase	1.00	0.96	6, 490	7	30.39
1	EA674_03595	Multidrug efflux transporter AdeK	1.00	1.00	883	5, 474	30.17
1	EA674_11070	OXA-51 family beta-lactamase	1.00	1.00	2, 268	9, 618	23.07
1	EA674_00940	Carbapenem susceptibility porin CarO	1.00	1.00	2, 742	1, 332	13.49
2	EA665_008865	OXA-51 family beta-lactamase	1.00	1.00	3, 386	3, 542	1.58
2	EA743_11455	Recombinase RecA	1.00	1.00	944	241	19.48
2	EA743_11495	Outer membrane protein BamA	1.00	1.00	5, 350	3, 679	10.96
2	EA743_11530	30S ribosomal protein RimO	1.00	1.00	2, 996	2, 037	10.63
2	EA743_11500	RIP metalloprotease RseP	1.00	1.00	1, 935	1, 305	9.81
2	EA743_11490	OmpH family outer membrane protein	1.00	1.00	1, 691	1, 161	9.71
3	EA667_019445	OXA-51 family beta-lactamase	1.00	1.00	9, 710	5, 192	11.24
4	EA719_004515	Carbapenem susceptibility porin CarO	1.00	0.62	4, 926	100	45.44

Additionally, a glutathione S-transferase family gene (EA674_008405) and the carbapenem susceptibility porin *carO* (EA674_000940) gene were highly expressed in TG22182 (R) in comparison to TG22627 (I). Both of these genes have been shown to confer resistance to beta-lactams and specifically carbapenems (100). Both isolates in Pair 1 also contain an OXA-51 beta-lactamase gene (*bla*_OXA−51_) (EA674_011070), although the gene is slightly up-regulated (4.5x) in TG22627 (I). TG22627 also showed higher expression of other genes associated with AMR including *adeI* (EA674_003605) (11x), *adeB* (EA674_009735) (9.5x), *adeA* (EA674_009730) (9.6x), and *adeJ* (EA674_003600) (9.6x); however, the up-regulation of genes in the AdeABC pump have previously been demonstrated to not confer aminoglycoside resistance (23).

These results suggest that the action of the PER-1 beta-lactamase has substrate specificity to ceftriaxone (CRO) and ceftazidime (CAZ). The PER-1 beta-lactamase protein has been associated with virulence in *A. baumannii* (101) and has also been associated with resistance to beta-lactams (102). However, the increased resistance of TG22627 to imipenem is likely due to the up-regulation of efflux-associated genes, as has been demonstrated previously (103).

#### Pair 2

The two pair 2 strains (TG3103, TG31986) showed variable resistance to cefepime (FEP), cefuroxime (CXM), and ceftriaxone (CRO). Twenty-four differentially-expressed regions were observed between Pair 2 isolates at a Wald stat value of 10 (Table S8). None of these regions were associated with known mechanisms of AMR in *A. baumannii*. The two isolates contained a class-D beta-lactamase (*bla*_OXA−51_) and a class-C beta-lactamase gene (*ampC*). There was no significant difference in gene expression of these regions between isolate pairs.

The composition of each beta-lactamase was determined at the nucleotide and peptide level. A protein alignment of *ampC* between TG31302 (I) and TG31986 (R) revealed a single amino acid difference (R to G) at position 172 in the PAZ domain. This non-synonymous mutation falls within the second of three characteristic conserved motifs, RxY^150^xN, for all class C serine beta-lactamase sequences (104). Although this mutation has not been previously associated with increased activity or misfolding of the protein, other mutations in *ampC* gene have (105), suggesting that this mutation may confer increased hydrolyzing activity against beta-lactams, although additional validation work is required to test this hypothesis.

A continuous stretch of 17 genes was upregulated in TG31986 (R) (EA743_011445 - EA743_011530) (Table S8). Nine genes within this region were in the top 30 differentially expressed genes in this analysis, including: recombinase *recA* (EA743_011455), outer membrane protein assembly factor *bamA* (EA743_011495), 30S ribosomal protein S12 methylthiotransferase *rimO* (EA743_011530), UMP kinase gene *pyrH* (EA665_011490), RIP metalloprotease *rseP* (EA743_011500), a gene coding for an OmpH family outer membrane protein (EA743_011490), a phosphatidate cytidylyltransferase gene (EA665_011475), a 1-deoxy-D-xylulose-5phosphate reductoisomerase gene (EA665_011470), and a di-trans,poly-cis-decaprenylcistransferase gene (EA665_011480). The outer membrane protein (OMP) H, a homolog of the Skp protein in *E. coli* (106), has been classified as a chaperone protein involved in the folding of BamA. Previous research has shown a correlation between upregulation of molecular chaperones when exposed to antimicrobials and the bacterium's improved ability to tolerate antimicrobial stress (107). Researchers have demonstrated that the *skp* gene in *E. coli* is an important stress-associated gene (108) that may be associated with AMR (109).

#### Pair 3

Pair 3 genomes (TG31307, TG29392) demonstrated variable resistance to cefepime (FEP), cefuroxime (CXM), ceftriaxone (CRO), and ceftazidime (CAZ). Of 94 genomic regions that were significantly differentially expressed (Walt stat of 10) (Table S9) between this isolate pair, one of the significant differences was between a *bla*_OXA−51_ family beta-lactamase gene (EA667_019445), which showed 1.9x up-regulation in the resistant strain (Table 5). Interestingly, 79 bases separated the end of insertion element IS*Aba1* and the start codon of *bla*_OXA−51_ in sample TG29392 (R); based on MinION sequencing, this version of *bla*_OXA−51_ is present on a plasmid in TG29392 (CP039931.1) and on the chromosome in TG31307. Previous research has demonstrated that this *bla*_OXA−51−like_ gene is ubiquitous across *A. baumannii* lineages, but only genomes containing the IS*Aba1* directly upstream of *bla*_OXA−51_ show resistance to carbapenems. It is likely that IS*Aba1* is acting as the promoter for *bla*_OXA−51_ in TG29392, conferring resistance to carbapenems (22). The intermediate resistance strain, TG31307, has both IS*Aba1* and *bla*_OXA−51_; however, IS*Aba1* is downstream of *bla*_OXA−51_ and therefore not functioning as a strong promoter for the beta-lactamase gene (Figure 3B). An analysis of the coding region of the *bla*_OXA−51_ gene in both isolates revealed no differences, suggesting that differences in resistance are likely due to gene expression.

Interestingly, several of the genes that were differentially expressed in the resistant strain in Pair 2 were up-regulated in the intermediate Pair 3 strain (TG31307). For example, *recA* was the most differentially expressed gene in Pair 3 genomes, but was up-regulated in TG31307 (Table S9). This suggests that the mechanisms of resistance have complex interactions that need to be investigated through the identification of epistatic interactions.

#### Pair 4

Susceptibility differences were observed between Pair 4 genomes for cefepime (FEP), gentamicin (GEN), and azithromycin (AZM). Of the 195 differences in gene expression (Wald stat of 10) between the resistant (TG22653) and intermediate strain (TG60155), perhaps the most striking is in the expression of *carO* (Table 5) (EA719_004515), an outer membrane porin (Table S10). Previous analyses have demonstrated that insertion sequences that disrupt *carO* are associated with decreased activity against beta-lactams (110). The genome assembly of the intermediate strain, TG60155, shows that *carO* is interrupted by the insertion sequence, IS*Aba1* (EA720_015165) (Figure 3C). An analysis of the transcriptome of TG60155 also failed to identify an intact *carO* transcript, which was present in TG22653.

### Antimicrobial Resistance Gene Expression Induction

In an effort to observe induced antimicrobial resistance in the four paired isolates, resistant strains were grown in sub-inhibitory concentrations of select antimicrobials. Differential expression of each sub-inhibitory isolate was compared to the isolate grown under inhibitory concentrations using the Wald statistic produced from DESeq2. Additionally, the resistant isolate TG22653 was grown under two different sub-inhibitory concentrations of cefepime (16 and 258 μg/mL) and differential expression was compared. No significant differential expression was observed in the four analyses based on an FDR-adjusted *p*-value of 0.05. Likewise, using the Wald statistic from these analyses also failed to identify significant differential expression between the differing antimicrobial concentrations using the chosen threshold. This suggests that differential expression is due to constitutively expressed mechanisms that are not inducible.

### AmpSeq Validation

Amplicon sequencing was performed on cDNA to not only confirm the RNA-Seq results, but also to provide a proof of concept as an advanced AMR diagnostic. Comparative expression was identified through comparison of ratios of the number of read counts of each targeted gene compared to a housekeeping gene (IX87_18340); the housekeeping gene, an ABC transporter permease, was identified as a gene with consistent, and relatively high, expression in the RNA-Seq data. For pair 1, the *bla*_PER−1_ and aphA1 genes were significantly upregulated in the resistant strain compared to the intermediate strain (Table 6). For pair 2, the expression of the *ampC* gene was not significantly different, suggesting that differential expression of this region doesn't fully explain the resistance phenotype and is consistent with the RNA-Seq data. For pair 3, the *bla*_OXA−51_ gene was confirmed to be significantly (*p* = 0.0003) up-regulated in the resistant strain compared to the intermediate strain. For pair 4, the *carO* gene was significantly (*p* < 0.0001) up-regulated in the resistant strain, which is consistent with the RNA-Seq results and is likely the primary mechanism of resistance. A gene associated with a chaperone-usher pili (CsuA/B) was highly up-regulated in the resistant strain. While not directly associated with AMR, this validation demonstrates consistency between the AmpSeq and RNA-Seq data.

**Table 6:** Differences in AmpSeq count data between resistant (R) and intermediate (I) isolate pairs.

**Pair**	**Locus**	**Read counts (R)**	**Read counts (I)**	**Houskeeping counts (R)**	**Housekeeping counts (I)**	**AVG. delta (R)**	**AVG. delta (I)**	**delta-delta**	***p*-value**
1	PER-1 (EA714_008075)	30, 867	309	30, 007	62, 187	1, 549	61, 878	60, 329	<0.0001
1	aphA1 (EA674_13195)	9, 172	9	30, 007	62, 187	23, 832	62, 178	38, 346	<0.0001
2	*ampC* (EA743_05675)	48, 550	45, 017	10, 047	10, 783	39, 002	34, 233	4, 769	0.240
3	OXA_65 (EA746_016395)	22, 818	24, 152	30, 446	40, 298	7, 078	16, 146	9, 068	0.0003
4	*carO* (EA719_004515)	31, 632	25, 088	7, 067	30, 654	20, 926	5, 566	15, 360	<0.0001
4	CsuA/B (EA719_006180)	23, 920	4	7, 067	30, 654	15, 309	30, 650	15, 341	<0.0001

### General Transcriptome Screen

A LS-BSR analysis of genes in the CARD database between the genome and transcriptome demonstrated that some genomic regions, such as the *adeF* gene (EA677_11720), were highly conserved in the genome, but were largely absent from the transcriptome (Table S11). Other genes associated with efflux, including *adeA* (EA679_RS16030), *adeH* (EA677_20370), *adeG* (EA686_07885), and an MFS transporter permease (EA708_RS14915) were also not expressed in laboratory growth conditions. These findings demonstrate the importance of incorporating gene expression when trying to understand phenotypic differences.

## Discussion

Antimicrobial resistance (AMR) is a significant, emerging threat, with *A. baumannii* being recently classified as a priority 1 pathogen (2). Some mechanisms associated with AMR in *A. baumannii* are understood, especially with regards to documented beta lactamases (111–114) and efflux pumps (15, 115, 116). However, the highly plastic pan-genome of *A. baumannii* (45) suggests that the identification of single, universal AMR mechanisms may be unlikely for some drugs. This same trend has been observed in other species with highly plastic genomes, such as *Pseudomonas aeruginosa* (117), and complicates surveillance and targeted therapy efforts. As such, *A. baumannii* is not only an emerging threat, but represents a critical challenge to the development of both novel drugs and molecular diagnostics.

In this study, we sequenced 107 genomes reported to be *A. baumannii* based on testing in the clinical laboratory. Typing based on WGS analyses identified 23 of the genomes were misclassified and belonged to other *Acinetobacter* species (Table S1). These incorrect clinical laboratory typing results highlight the need for improved clinical diagnostics of *A. baumannii*. An additional 35 genomes in the GenBank assembly database were incorrectly annotated as *A. baumannii* and belonged to other species (not shown), which further demonstrates the difficulty in typing as well as and the impact of mis-annotated genomes on population structure analysis in *Acinetobacter*. Typing strains using WGS should be a first step in any large comparative genomics study to limit the analysis to a targeted group, clade, or species.

We generated antibiograms for 12 drugs, representing multiple drug families, across the 84 isolates confirmed to belong to *A. baumannii* by WGS analysis. Some of the drugs screened in this study aren't typically used in current treatment regimens for *A. baumannii* infections. However, with growing resistance emerging to next generation drugs, clinicians are exploring older drugs (e.g., chloramphenicol) to treat emerging threats (42, 118). In this study, we sought to identify genomic differences that could explain the variable resistance phenotypes using established antimicrobial resistance gene databases as a method to predict AMR from genomics data (31, 32, 35, 68). A screen of regions from the Comprehensive Antimicrobial Resistance Database (CARD) against genomes sequenced in this study failed to identify characterized resistance mechanisms that largely explain the resistance phenotype. These results demonstrate the limitations to this approach in highly plastic species and suggest that alternative approaches, including RNA-Seq, may be required for a comprehensive understanding of AMR mechanisms in *A. baumannii*.

We then employed reference independent, bacterial genome wide association study (bGWAS) methods to identify genomic differences between susceptible/intermediate/resistant phenotypes. These types of associations have been used in other pathogens to identify genotype/phenotype associations (119). In general, we failed to identify a clear association between the genotype (21 bp Kmers, coding regions, SNPs) and the resistance phenotype when comparing either all *Acinetobacter* or just *A. baumannii* genomes (Table 3) without accounting for population structure. Methods that do incorporate population structure using SNPs (treeWAS) or Kmers (pyseer) identified associations for some drugs and could be the focus of additional work. One limiting factor in bGWAS studies is the number of samples in each group. While our study also suffered from small sample sizes, this work provides a framework for future bGWAS studies in *A. baumannii*.

Recent research has demonstrated the difficulty in identifying complex mechanisms, or under-or-over-represented phenotypes, using a GWAS approach (120). As a way to focus on sparsely distributed AMR mechanisms, a paired isolate approach was utilized in order to reduce noise in the genomic background. In this analysis, four isolate pairs were individually compared across four antimicrobials (Table 2). RNA-Sequencing (RNA-Seq) of these four paired resistant/intermediate isolates that shared a common genetic background was employed. This paired approach identified several novel and previously identified mechanisms, but also highlights the difficult in identifying mechanisms through standard comparative genomics approaches. While known resistance mechanisms were identified in the resistant strains, some of those regions were also identified in intermediate strains; previous studies of *A. baumannii* transcriptomes have also observed up-regulation of resistance and efflux genes in susceptible strains (5). RNASeq data allowed for the identification of antimicrobial resistance mechanisms that while present in both the resistant and susceptible genomes, were differentially expressed due to an upstream insertion element. These results also highlight the possibility that expression of a single AMR gene does not always confer resistance and it is likely that combinations of genes are responsible for observed resistance. Differential expression differences were confirmed using a cDNA-AmpSeq approach and largely confirmed the differential expression of targeted regions. Previous research has demonstrated a bias of differentially expressed regions when applying a multiplexed PCR approach (121). We addressed this issue by optimizing primer concentrations using genomic DNA and including a single copy number gene for normalization.

The results of this study demonstrate that, due to the plastic nature of *A. baumannii's* pan-genome, comprehensive AMR surveillance cannot solely be achieved through genomics methods alone, especially with current AMR databases and commonly used analytical methods. This study demonstrates that AMR genes are not conserved across *A. baumannii* lineages with similar AMR profiles and that solely relying on genomics methods for AMR surveillance and discovery, such as gene presence/absence, will fail to detect novel or recently acquired AMR mechanisms. For instance, identifying only the position of insertion sequence (IS) elements throughout a genome using informatics tools provides little resolution in the identification of AMR genes upregulated by the presence of upstream IS elements. However, by utilizing transcriptome data, AMR genes upregulated by these elements as well as novel AMR mechanisms were identified; these targets were used to design cDNA amplicon sequencing targets for these mechanisms to improve surveillance and diagnostic efforts.

## Data Availability Statement

The datasets generated for this study can be found in the PRJNA497581.

## Ethics Statement

Ethical approval for this study was not required in accordance with local legislation and national guidelines. All human-derived samples were collected under ethics protocols from clinical laboratories. Patients included in this study were anonymized and no written informed consent was acquired because of the retrospective nature of the study.

## Author Contributions

CR performed laboratory experiments, analyzed data, and helped to write the manuscript. CW and KK helped to write the manuscript. AV performed laboratory experiments. MV and VH performed laboratory analyses. JB and ED was involved with experimental design. PP was involved in data analysis. DE was involved in experimental design. JS was involved with experimental design, analyzed data, and helped to write the manuscript. All authors contributed to the article and approved the submitted version.

## Conflict of Interest

The authors declare that the research was conducted in the absence of any commercial or financial relationships that could be construed as a potential conflict of interest.

## Supplementary Material

Figure S1 A maximum-likelihood phylogeny of global *A. baumannii* genomes inferred from an alignment of 11,687 concatenated SNPs. Red dashes indicate genomes sequenced in this study.

Figure S2 A maximum-likelihood phylogeny of paired isolates. The conservation of selected proteins from the CARD database, based on blast score ratio (BSR) values, is shown as a heatmap. The BSR values were visualized with the Interactive tree of life (91).

Figure S3 A maximum likelihood phylogeny of select *A. baumannii* genomes. The distribution of the *bla*_PER−1_ beta-lactamase gene in ST368 genomes, based on blast score ratio (BSR) values, was visualized as a heatmap with the Interactive tree of life (91).

Table S1 Metadata for genomes sequenced in this study.

Table S2 MIC values for screened antimicrobials across all genomes.

Table S3 PCR primers for targeted amplicon sequencing.

Table S4 BSR values for differentially-present CARD proteins.

Table S5 The distribution of CARD genes against paired isolate genomes.

Table S6 bGWAS associations between resistant and susceptible phenotypes. Differences in Kmer frequences were identified with a Mann-Whitney test with Bonferroni correction.

Table S7 DESeq2 results of Pair 1 RNA-Seq datasets. Biological replicates denoted with (a,b,c). Isolates grown in sub-inhibitory concentrations are labeled as “sub” with the corresponding antimicrobial.

Table S8 DESeq2 results of Pair 2 RNA-Seq datasets. Biological replicates denoted with (a,b,c). Isolates grown in sub-inhibitory concentrations are labeled as “sub” with the corresponding antimicrobial.

Table S9 DESeq2 results of Pair 3 RNA-Seq datasets. Biological replicates denoted with (a,b,c). Isolates grown in sub-inhibitory concentrations are labeled as “sub” with the corresponding antimicrobial.

Table S10 DESeq2 results of Pair 4 RNA-Seq datasets. Biological replicates denoted with (a,b,c). Isolates grown in sub-inhibitory concentrations are labeled as “sub” with the corresponding antimicrobial. Two different concentrations of cefepime (FEP) were used to test induction (16, 128).

Table S11 A BSR matrix of CARD genes in the genome and transcriptome of each paired isolate.
