# Supplementary figures and images for: Bacterial Genome Wide Association Studies (bGWAS) and Transcriptomics Identifies Cryptic Antimicrobial Resistance Mechanisms in Acinetobacter baumannii
Source: Front Public Health. 2020 Sep 2;8:451. doi: 10.3389/fpubh.2020.00451 (PMC7493718; doi:10.3389/fpubh.2020.00451)

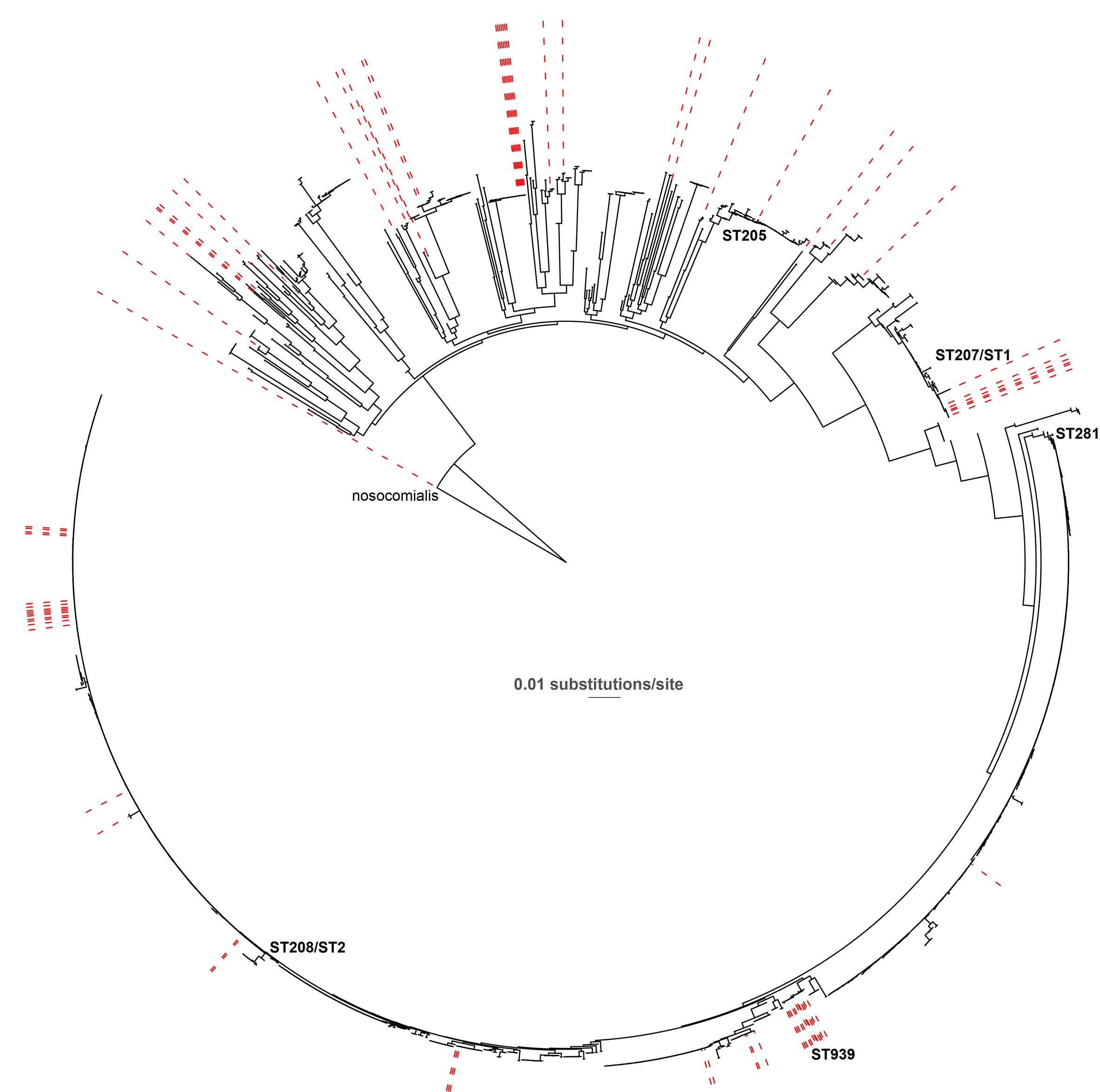

Supplement: Figure S1 — A maximum-likelihood phylogeny of global A. baumannii genomes inferred from an alignment of 11,687 concatenated SNPs. Red dashes indicate genomes sequenced in this study. [file Image_1.JPEG]

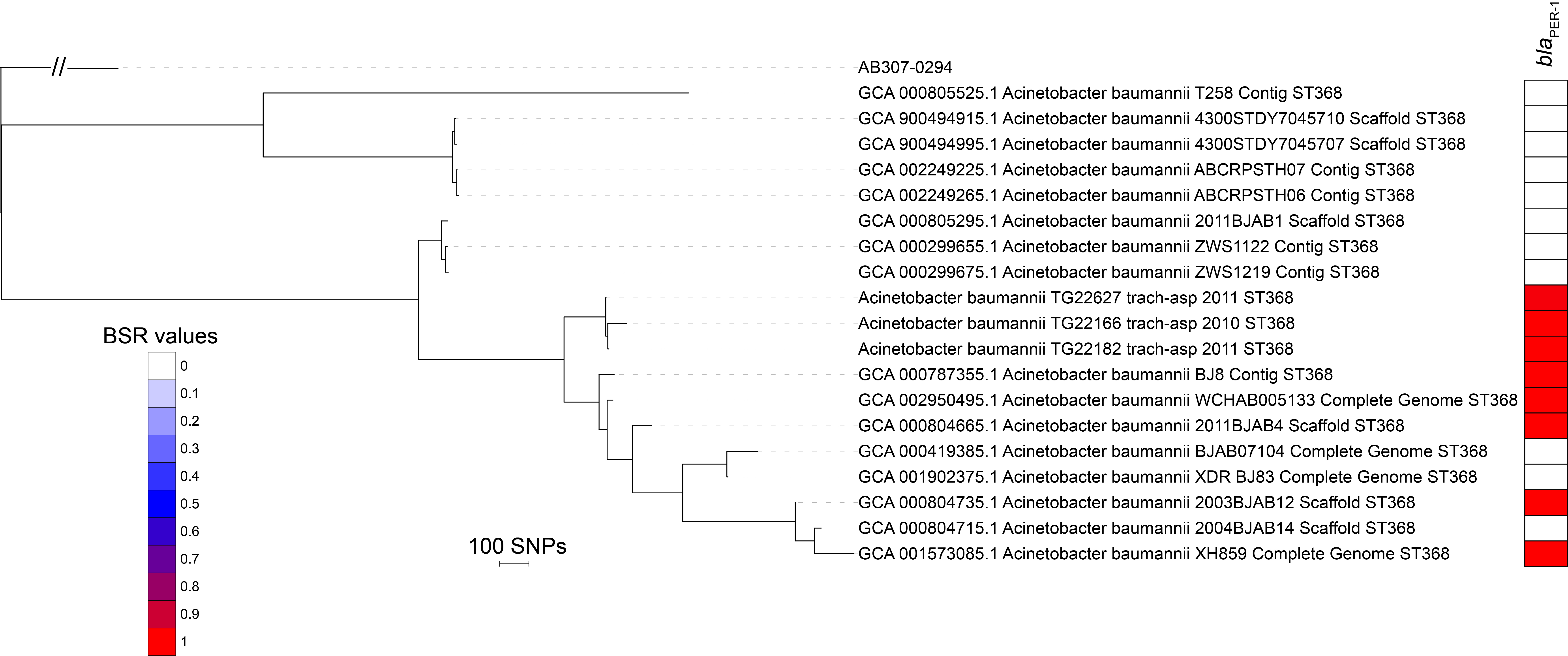

Supplement: Figure S3 — A maximum likelihood phylogeny of select A. baumannii genomes. The distribution of the blaPER−1 beta-lactamase gene in ST368 genomes, based on blast score ratio (BSR) values, was visualized as a heatmap with the Interactive tree of life (91). [file Image_3.JPEG]
